# Super-selective arterial embolization in the therapy of non-ischemic priapism—a single-center study and literature review

**DOI:** 10.1186/s42155-026-00672-0

**Published:** 2026-03-18

**Authors:** Carolina Dominguez Aleixo, Maximilian de Bucourt, Maximilian Lindholz, Markus Lerchbaumer, Uli Fehrenbach, Federico Collettini, Bernhard Gebauer, Julian Lenk, Timo Alexander Auer

**Affiliations:** 1 Department of Radiology, Charité – Universitätsmedizin Berlin, Corporate member of Freie Universität Berlin and Humboldt-Universität zu Berlin, Berlin, Germany; 2 Berlin Institute of Health (BIH), Berlin, Germany

**Keywords:** Priapism, Interventional radiology, Angiography, Arterial embolization

## Abstract

**Background:**

The goal of our analysis is to provide technical information and clinical long-term data on arterial embolization for non-ischemic priapism. Furthermore, this study presents a comprehensive literature review.

**Methods:**

We analyzed patient data from June 2005 to June 2025 at a large university hospital, focusing on patients with non-ischemic priapism lasting over 1 week, unresponsive to conservative treatment, and referred for arterial embolization. Age, symptom etiology, initial diagnostic modality, embolic agent, uni- or bilateral arterial supply of the fistula/pseudoaneurysm, technical success, clinical outcome, need for a second attempt, erectile dysfunction, adverse events, and mean follow-up time needed were assessed. Findings were contextualized with studies from the past two decades.

**Results:**

A total of 15 male patients with non-ischemic priapism due to blunt, penetrating trauma or of idiopathic origin, were included in this analysis. The embolic agents chosen included gelatin sponge, polyvinyl alcohol particles, autologous clot, microcoils, and a combination of microcoils with gelatin sponge or polyvinyl alcohol particles. Technical success was achieved in 14 patients (93.3%). A second or third intervention was needed in three cases (20.0%) to achieve clinical success. Documented adverse events included procedure-related findings such as penile skin changes and deviation in two patients (13.3%). No new cases of erectile dysfunction were reported (0%).

**Conclusion:**

Our findings support super-selective arterial embolization as a safe, technically successful, and minimally invasive therapy option for non-ischemic priapism after conservative measures fail. Moreover, our data suggest that arterial embolization is associated with long-term symptom improvement without significantly impairing sexual function.

**Graphical Abstract:**

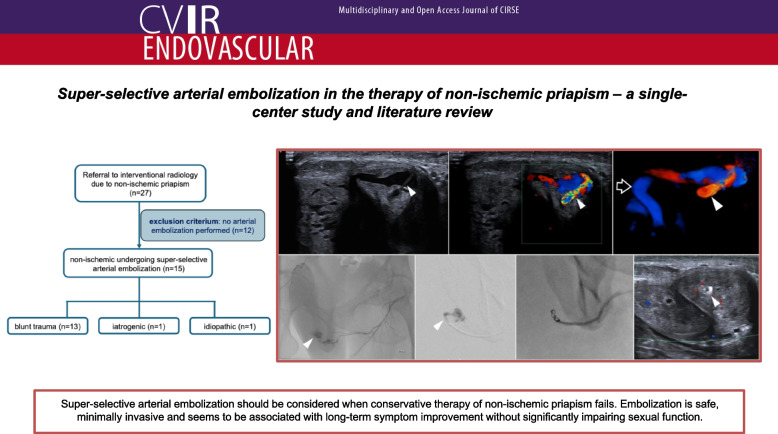

**Supplementary Information:**

The online version contains supplementary material available at 10.1186/s42155-026-00672-0.

## Background

Priapism is a pathological condition characterized by a persistent, unwanted penile erection without sexual stimulation [1]. According to etiology and cavernous blood gas analysis, priapism is differentiated into ischemic priapism, a urological emergency, distinguished by a localized compartment syndrome due to blood accumulation in the cavernous bodies [1, 2] and non-ischemic priapism, a rare state that arises predominantly from blunt injuries [3]. The diagnosis of penile vascular damage is routinely performed by color-coded Doppler ultrasound with Duplex measurements [4, 5]. Standard conservative therapies include compression of the perineum, local ice application, and watch-and-wait [6]. Non-ischemic priapism commonly spontaneously resolves [7, 8]. In case of conservative treatment failure and absent spontaneous resolution, selective arterial embolization plays a key role. Embolization therapy is usually individualized according to penile injury characteristics and the experience of the interventional radiologist with certain embolic agents [9]. The main discussed possible adverse event is new erectile dysfunction (ED) [10]. Based on the five-item version of the International Index of Erectile Function (IIEF-5) questionnaire, five categories of ED may be distinguished according to final scores: no ED (22 to 25), mild (17 to 21), mild to moderate (12 to 16), moderate (8 to 11), and severe ED (5 to 7) [11]. Since non-ischemic priapism is rare and often conservative measures suffice, data on timing, choice of embolic agent, and technical details of embolization as a therapeutic option lack [12]. The purpose of our analysis is to increase knowledge on arterial embolization as a therapy option for non-ischemic priapism following unsuccessful conservative measures while preserving baseline urogenital function.

## Methods

**Fig. 1:**
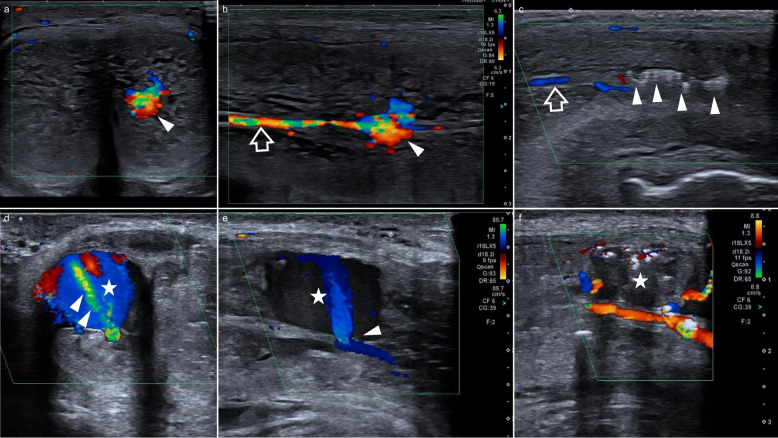
Color-coded Doppler ultrasound of a small arterio-cavernous fistula on the left cavernous body (arrowhead, **a** + **b**) with successful coil embolization (**c**, arrowhead) and denotable change of arterial inflow after treatment (arrow, **b** + **c**). In contrast, large right-sided arterio-cavernous fistula (asterisk, **d**–**f**) with fanfare-like inflow (arrowhead, **d** + **e**) and successful embolization with gelatin (**f**). Image orientation: **a**, **d**: transversal plane; **b**, **c**, **e**, **f**: longitudinal plane

This retrospective study involving human participants is in accordance with the ethical standards of the institutional and national research committees, as well as the 1964 Helsinki Declaration and its subsequent amendments. The Human Investigation Committee (IRB) of Charité – University Medicine Berlin approved this study (EA1/164/25). Data on patients referred to interventional radiology for super-selective arterial embolization in the therapeutic management of priapism were retrieved from digital files between June 2005 and June 2025. All patients with priapism refractory to conservative measures and lasting for more than 1 week were initially selected. On physical examination, patients had a persistent semirigid penis. Only those with a clinical diagnosis of non-ischemic priapism who underwent selective arterial embolization following multidisciplinary decision-making were included. Age, symptom etiology, initial radiological diagnostic modality (Doppler ultrasound with or without additional CT or MRI; Fig. 1 and an Additional file 1 show this in more detail), uni-/bilateral arterial supply of the fistula or pseudoaneurysm on angiography, embolic agent(s), technical success, clinical outcome, need for an additional attempt, procedure-related adverse effects, and mean follow-up time needed (in months) were analyzed. ED was assessed at least 4 months after the procedure using IIEF-5. In patients with an IIEF-5 score below 22, erectile function was additionally determined retrospectively for the symptomatic period before the intervention.

A fistula was diagnosed on ultrasound or contrast-enhanced CT based on early venous filling or direct visualization and confirmed on angiography by arteriovenous shunting with rapid contrast of the draining vein. A pseudoaneurysm was identified as a contrast-filled sac connected to the artery on contrast-enhanced CT-angiography, as a yin–yang Doppler flow pattern, and/or as a focal outpouching with persistent contrast pooling on angiography. Arterial trauma was defined by imaging evidence of vessel irregularity, dissection, abrupt cutoff, contrast extravasation, or stenosis, demonstrated angiographically.

**Fig. 2:**
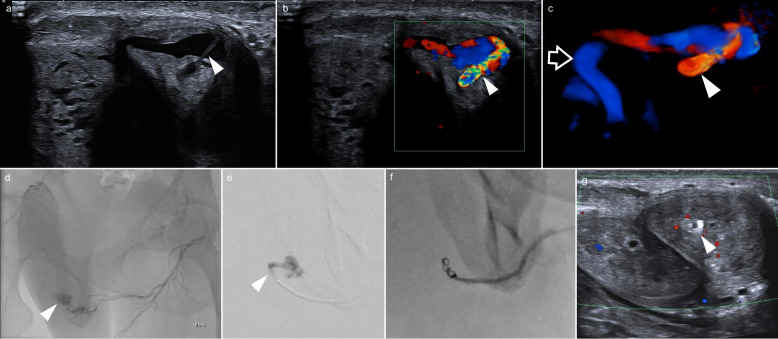
Representative case of super-selective angiographic embolization of a chronic arterio-cavernous fistula on the left cavernous body visualized on US (arrowhead, **a**–**c**) with a large draining vein (arrow). Embolization (**d**, **e**) with super-selective targeting of the fistula (**e**) and corresponding coil embolization (**f**). Successful intervention and coil localization depicted on US 1 day after intervention (**g**)

All interventions were performed by interventional radiologists with 3 or more years of experience in embolization techniques; embolic agents were chosen based on individual experience. Technical success was defined as complete angiographic absence of fistula or pseudoaneurysm after embolization (illustrated example in Fig. 2). Clinical outcome was positive when cessation of priapism was achieved after the intervention (after up to three attempts). Constant ED was defined as an IIEF-5 score below 22 and/or persisting symptoms of unsatisfactory sexual function both before and after the intervention, and new ED was defined as a decrease in IIEF-5 score below 22 only after embolization. ED was differentiated into transient or persistent, depending on symptom duration of less than or more than 3 months after the procedure, respectively [13]. Procedure-related adverse effects included puncture-site pain or hematoma, penile skin changes, penile deviation, or new ED. Post-procedural follow-up was either conducted through urological consultations or performed by interventional radiology via standardized telephone interviews, focusing on clinical success, complications, and erectile function (including IIEF-assessment where available). The need for urological follow-ups beyond 4 months post-intervention was documented as long-term follow-up.

To contextualize findings, a manual PubMed search was conducted to identify and include studies published between 2003 and 2025 that reported cohorts of eight or more patients undergoing super-selective embolization for non-ischaemic priapism. Keywords included “non-ischemic priapism,” “arterial embolization and priapism,” “arterio-cavernous fistula,” and “high-flow priapism and interventional radiology.” Outcomes were defined according to the same criteria used in our analysis, ensuring consistency between results and their definitions. Titles and abstracts of the studies were analyzed for relevance, followed by a full-text review of potentially suitable articles. Only studies with extractable data relevant to the procedure and clinical outcomes were included.

To improve the readability of the manuscript, Grammarly, version 1.146.3.0, was used on December 25, 2025. Drs. Dominguez Aleixo and Lenk take responsibility for the integrity of the generated content.

### Statistical analysis

Continuous variables are expressed as medians with interquartile ranges (IQRs) and categorical variables as counts with percentages. The frequency of repeat procedures was categorized as a discrete ordinal variable with three levels (one, two, or three procedures), and results are shown as counts with percentages. No inferential statistical tests were conducted due to the limited sample size. Analyses were conducted using R Version 4.3.3 (The R Project for Statistical Computing).

## Results

### Baseline characteristics

**Fig. 3:**
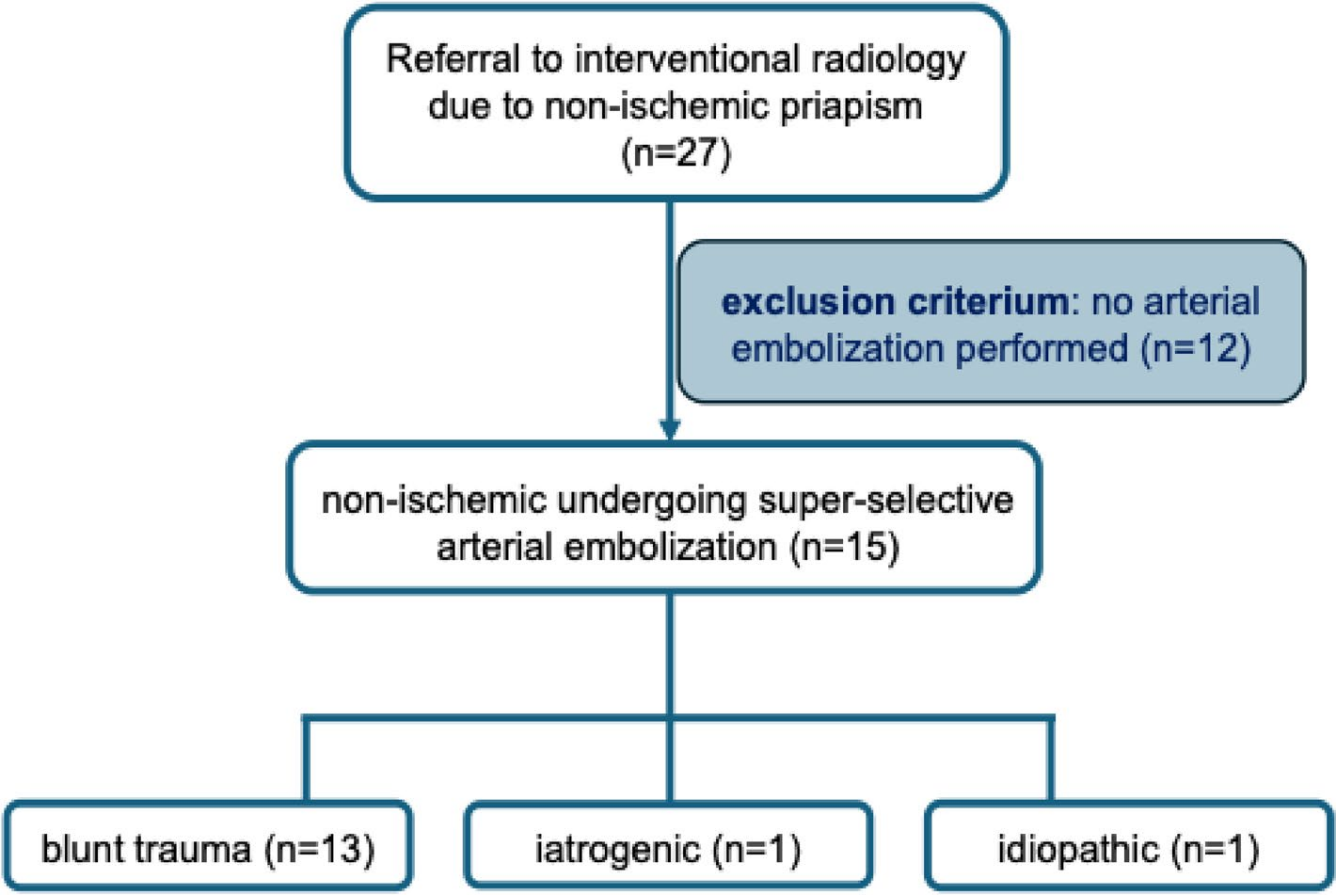
Flowchart illustrating patient inclusion and exclusion

**Fig. 4:**
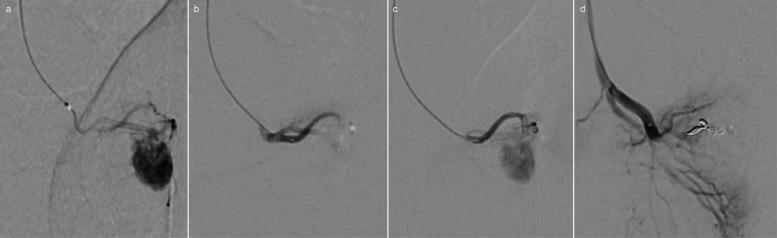
Arterio-cavernous fistula before (**a**) and after (**b**) embolization with the temporary agent, followed by reperfusion (**c**) and embolization (**d**) with the permanent agent – modified image based on (Aleixo, 2025) [14]

**Fig. 5:**
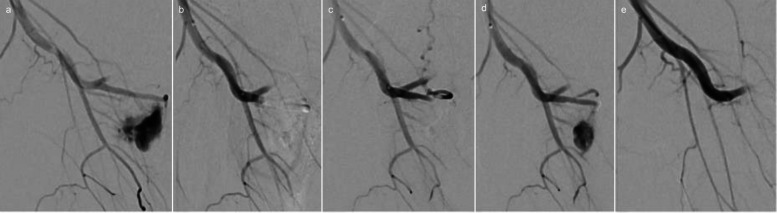
Arterio-cavernous fistula before (**a**) and after (**b**) embolization with the temporary, less viscous gelatin sponge, followed by slow (**c**) and fast (**d**) reperfusion and final embolization with the temporary, more viscous gelatin sponge (**e**)

A total of 15 male patients with a median age of 21 years (IQR 10.5–31.1) at the time of embolization were included in this analysis. From the 27 patients considered initially in multi-disciplinary decision-making, 12 did not undergo embolization and were excluded. All included patients presented with priapism refractory to conservative measures, namely local pressure or cryotherapy. Etiology was most commonly blunt penile trauma, mainly when bi-/motorcycling, occurring in 13 patients (86.7%). Iatrogenic injury, following penile injection, accounted for the etiology in one patient (6.7%). One patient had no identifiable cause (6.7%), rendering it idiopathic (Table 1 for demographics and patient characteristics and Fig. 3 for patient selection and etiologies). Doppler ultrasound was the primary diagnostic modality in 13 patients (86.7%). Additional CT was performed in two patients (13.3%) and MRI in one patient (6.7%). Two patients (13.3%) were diagnosed according to cavernous blood gas analysis only. Angiography revealed right-sided cavernous artery involvement in six patients (40.4%), left-sided involvement in five patients (33.3%), and bilateral involvement in four patients (26.7%). Embolic agents included polyvinyl alcohol (PVA) particles, autologous blood clot, gelatin sponge, microcoils, or two of these agents combined (microcoils in combination with either particles or gelatin sponge). For the initial therapeutic embolization, gelatin sponge was chosen in five cases (33.3%), microcoils in five cases (33.3%), PVA-particles in one case (6.7%), a combination of gelatin sponge and microcoils in three cases (20.0%), and PVA-particles with microcoils in one case (6.7%). In two cases (13.3%), embolic agents were adjusted during the procedure due to immediate insufficient success: in one case, failure of gelatin sponge led to the choice of coil-embolization to achieve technical success during the same session (example illustrated in Fig. 4); in one additional case (6.7%), increased viscosity of gelatin sponge was needed after insufficiency of a less viscous dilution (Fig. 5).

**Table 1:** Demographics and outcomes

		patients (***n*** = 15)
Age, years (median (IQR))		21.3 (10.5–31.1)
Primary diagnostic modality (*n* (%))	Doppler ultrasound	13 (86.7)
	none	2 (13.3)
Embolized side (*n* (%))	Right	6 (40.4)
	Left	5 (33.3)
	Both	4 (26.7)
First attempted embolic agent (*n* (%))	Gelatin sponge	5 (33.3)
	Autologous clot	0 (0)
	Particles	1 (6.7)
	Microcoils	5 (33.3)
	Combination^a^	4 (26.7)
Second or third attempt needed for clinical success (*n* (%))		3 (20.0)
Technical success (*n* (%))		14 (93.3)
Long-term follow-up needed (*n* (%))		4 (26.7)
Adverse effects (*n* (%))		2 (13.3)
Transient ED (*n* (%))		1 (6.7)
Constant ED (*n* (%))		4 (26.7)

Technical success is defined as complete angiographic absence of fistula or pseudoaneurysm after super-selective arterial embolization

*IQR* interquartile range, *CT* computerized tomography, *MRI* magnetic resonance imaging, *ED* erectile dysfunction. Data are presented as median (IQR) or *n* (%)

^a^Combination stands for gelatin sponge with microcoils or PVA particles with microcoils

### Outcome results

Technical success was achieved in 14 patients (93.3%). Three patients (20.0%) relapsed within 24 h after the first intervention and required one or two re-interventions, mostly due to a second feeder of the arterio-cavernous fistula not identified or visualized on initial angiography. In the first of these cases, gelatin sponge was chosen for the first attempt, followed by autologous clot in the successful second attempt. In the second case, embolization was first performed with gelatin sponge; in the second and third attempts, gelatin sponge was combined with coils. Coils were the chosen agent for the third case, also requiring a second and third attempt for resolution. Adverse effects, including short-term penile skin changes and/or long-term deviation of the penis to the affected side, were reported in two patients (13.3%). Four patients (26.7%) required long-term urological follow-up due to post-procedural adverse effects and/or transient ED requiring phosphodiesterase-5 (PDE5) inhibitors. Seven patients (46.7%) were lost to follow-up in interventional radiology. The median follow-up was 14 months (IQR 0–86), with a mean follow-up time of 32.4 months, and a maximum follow-up of 148 months. One patient (6.7%) developed transient ED after embolization. Post-interventional addition of PDE5 inhibitors was sufficient to achieve satisfactory erectile function in this patient; the IIEF-5 score at 1-year follow-up was 25. Among patients with follow-up in interventional radiology (53.3%), the median post-procedural IIEF-5 score was 23 (IQR 16.5–25). Persistent ED was documented in four patients (26.7%). Two of these patients reported constant mild and moderate ED (respectively) before and after embolization, while the remaining two with severe ED before the embolization presented with only mild or mild to moderate ED in the long-term follow-up. Three of the four patients with persistent ED underwent embolization with microcoils. The median age of patients with persistent ED was 38.5 years (IQR 27–46) at the time of the intervention. Etiology in this subgroup was blunt trauma (50%), followed by iatrogenic and idiopathic (25%, respectively). Among the seven patients (46.7%) lost to long-term follow-up in interventional radiology, no post-procedural ED was documented. Outcome details are illustrated in Table 2, and Additional file 2 shows long-term follow-up data in more detail.

**Table 2:** Patients requiring repeat embolization after relapse

Patient	Initial embolic agent	Second attempt agent	Third attempt agent	Technical success
1	Gelatin sponge	Autologous clot	-	Yes
2	Gelatin sponge	Gelatin sponge, coils	Gelatin sponge, coils	Yes
3	Coils	Coils	Coils	Yes

Technical success was defined as complete angiographic absence of fistula or pseudoaneurysm after super-selective arterial embolization

## Discussion

Our results show that super-selective arterial embolization is an effective and safe treatment option for non-ischemic priapism, particularly when caused by an arterio-cavernous fistula or pseudoaneurysm, and conservative measures fail. Technical success was achieved in nearly all patients, and clinical resolution occurred in most, with only a minority requiring repeated attempts. Adverse events were scarce or mild. Importantly, new ED was not observed during long-term follow-up, and post-procedure erectile function was stable or improved.

These findings support embolization as a minimally invasive therapy option with symptom resolution and preservation of sexual function. The need for procedure repetition likely reflects variations in vascular complexity rather than technical limitations. The absence of new ED cases in our cohort suggests that vessel selection and individualized choice of embolic agents may decrease procedure risks.

Non-ischemic priapism is not a urological emergency and rarely requires urgent treatment [6]. Conservative therapy should be the first-line approach, as it has shown to be sufficient, especially among pediatric patients [15]. The rarity of cases requiring endovascular therapy is reflected in the limited sample sizes across published studies. As summarized in Table 3, studies published between 2003 and 2025 include cohorts ranging from 8 to 27 patients, with a total of 153 patients. Blunt penile trauma, often from straddle injury during bi-/motorcycle accidents, represents the most common etiology, as observed in our cohort [3, 6]. Non-ischemic priapism may rarely develop from ischemic priapism. Occasionally, no cause is identified [26]. Laceration of the penile artery or its branches may result in an abnormal connection to the adjacent cavernous body, forming an arterio-cavernous fistula and/or pseudoaneurysm [27]. The constant arterial blood supply of the cavernous body leads to the prolonged, uncontrolled erection of the penis [27]. Delayed symptom onset may occur due to vasospasms within the initial hematoma [28]. As in our cohort, Doppler ultrasound remains the leading diagnostic approach [29, 30], with MRI and CT reserved for complex trauma [2, 31]. Arterial embolization enables precise occlusion of the vascular malformation with low recurrence rates, high technical success, and minimal invasiveness [21, 32]. Previous studies report success rates above 90%, irrespective of the embolic agent chosen [20, 32, 33]. Temporary agents are often preferred when bilateral embolization is indicated to reduce the risk of permanent ED, whereas permanent agents may provide lower recurrence rates at the potential risk of permanent vascular occlusion [19, 20, 34]. In our cohort, three patients required repeat embolization to achieve clinical success, while another three required adjustments of the embolic material during the procedure for technical success. Reported re-intervention rates to achieve clinical success vary from 0 to 44% [16, 25]. This discrepancy may reflect variations in embolization technique, operator experience, and patient-specific anatomical considerations, as well as definitions of clinical success and their verification and validation over time during follow-up. Importantly, no major adverse effects were reported across studies. Operator expertise is particularly important in complex cases, such as in bilateral arterial supply or failure of the initially chosen embolic agent, where the flexibility to switch strategies mid-procedure and individually tailor the embolization is crucial [9].

**Table 3:** Summary of clinical features and results from the selected studies

**Study**	Bertolotto et al. [16]	Savoca et al. [17]	Kim et al. [18]	Liu et al. [19]	Pei et al. [20]	Chick et al. [21]	De Magistris et al. [22]	Bi et al. [23]	Von Stempel et al. [24]	Biasatti et al. [25]
Year of publication	2003	2004	2007	2008	2018	2018	2020	2020	2023	2025
Patients (*n*)	9	15	27	8	16	20	9	14	22	13
Age, years (mean)	29	32	39	33	24	36	33	38	n/a	30
Primary diagnostic modality	Doppler ultrasound	Doppler ultrasound	Doppler ultrasound	Doppler ultrasound	Doppler ultrasound	Doppler ultrasound	Doppler ultrasound	Doppler ultrasound	Doppler ultrasound	Doppler ultrasound
Most used embolic agent	PVA-particles	PVA-particles	Gelatin sponge, autologous clot (individually)	Microcoils	Microcoils	Gelatin sponge	Microcoils	Gelatin sponge	Microcoils	Microcoils
Technical success (%)	100	93	100	100	100	n/a	100	100	n/a	100
Need for > 1 attempt to reach clinical success (%)	44	27	11	25	6	0	22	7	40	0
Adverse effects (%)	0	0	0	0	0	5	0	0	n/a	0
Persistent ED (%)	11	7	26	25	13	0	11	7	n/a	n/a

Data are presented as *n* (%)

*ED* erectile dysfunction, *IIEF* International Index of Erectile Function

ED remains the most relevant adverse effect. A 2-year review including 117 patients reported persistent ED in 15%, with the highest rates following microcoil embolization [9]. Our findings are consistent with those summarized in Table 3, which overall report relatively low rates of persistent ED, ranging from 0 to 26%, with most studies describing values under 15%. Notably, studies published after 2018 show lower ED rates compared to studies in the decade before this year, potentially reflecting advances in embolization techniques, improved material selection, particularly with the more widespread choice of microcoils and the associated growth in technical expertise with their use. Structured follow-up is needed, as ED may be influenced by vascular, neurogenic, and psychological factors [2, 35]. PDE5 inhibitors may be beneficial in transient cases [24, 36].

Limitations in our analysis restrict its generalizability. In line with current literature on this rare indication, an important limitation is the small cohort size and retrospective design. Formal statistical analyses with respective *p*-values were not performed due to insufficient event counts. Only four cases of persistent ED were documented, restricting robust statistical interpretation and generalization of findings. Assessment of baseline erectile function relied on retrospective self-reporting, introducing potential selection and recall bias. Another limiting factor is the young patient’s age at the time of the intervention, as ED is not a common concern in pediatric populations, and IIEF questionnaires are not applicable in this age group. The influence of symptom duration before embolization on outcomes remains unclear and warrants questioning in prospective, larger studies. Further potential confounding factors, such as patient age, baseline health status, and comorbidity burden, which could have influenced the observed outcomes independently of the intervention, should be included in future larger and adequately powered inferential analyses. Additionally, variability in operator experience and technical skill was not formally assessed and may have contributed to differences in procedural performance and outcomes. Finally, with 46.7% of patients lost to long-term follow-up, conclusions regarding post-procedural erectile function should be interpreted with caution, as the reduced follow-up rate limits the robustness of long-term functional outcome assessment.

## Conclusions

An individualized approach to super-selective arterial embolization in non-ischemic priapism is essential. Bilateral angiography should be performed to identify all arterial feeders and minimize repeat interventions. However, the chosen approach also depends on the operator’s experience and the quality of the prior ultrasound examination. When the side of the feeding artery is clearly localized and in very young patients, a unilateral examination may be sufficient to minimize radiation exposure. Embolic agent selection should consider patient age, baseline erectile function, and the interventionalist’s experience. There are no reliable data suggesting the superiority of any embolic agent. In a super-selective position, we recommend microcoils or a gelatin torpedo. We advise against liquid embolic agents and particles. Erectile function should be assessed at baseline and closely monitored in collaboration with urology during the first six post-interventional months. To identify and possibly treat adverse effects, new or persistent ED, a monthly follow-up may be needed in this period. Our findings, supported by over two decades of literature, indicate that embolization is a safe, effective, and minimally invasive treatment option for non-ischemic priapism refractory to conservative management, offering durable symptom resolution and a potentially favorable preservation of erectile function.

## Supplementary Information

Additional file 1: Representative contrast-enhanced CT images of an arterio-cavernous fistula (arrowhead) in the right cavernous body (sagittal [a], coronal [b], and transversal planes [c])– modified image based on (Aleixo, 2025) [2] – and T2-weighted MR imaging of an arterio-cavernous fistula in the left cavernous body in sagittal (d) and transversal plane (e).

Additional file 2: Patients with follow-up in interventional radiology.

## Data Availability

The datasets used and/or analyzed during the current study are available from the corresponding author on reasonable request.

## Abbreviations

ED: Erectile dysfunction
PVA: Polyvinyl alcohol
CT: Computerised tomography
MRI: Magnetic resonance imaging
US: Ultrasound
IIEF: International Index of Erectile Function
IQR: Interquartile range
PDE-5: Phosphodiesterase-5
