# Supplementary material for: Super-selective arterial embolization in the therapy of non-ischemic priapism—a single-center study and literature review
Source: CVIR Endovasc. 2026 Mar 18;9:30. doi: 10.1186/s42155-026-00672-0 (PMC13000060; doi:10.1186/s42155-026-00672-0)
Supplement: Supplementary file 1 — Additional file 1: Representative contrast-enhanced CT images of an arterio-cavernous fistula (arrowhead) in the right cavernous body (sagittal [a], coronal [b], and transversal planes [c])– modified image based on (Aleixo, 2025) [2] – and T2-weighted MR imaging of an arterio-cavernous fistula in the left cavernous body in sagittal (d) and transversal plane (e). [file 42155_2026_672_MOESM1_ESM.docx]

**Additional file 1** Representative contrast-enhanced CT images of an arterio-cavernous fistula (arrowhead) in the right cavernous body (sagittal [a], coronal [b] and transversal planes [c]) – modified image based on (Aleixo, 2025) [2] – and T2-weighted MR-imaging of an arterio-cavernous fistula in the left cavernous body in sagittal (d) and transversal plane (e).
