# Supplementary material for: Super-selective arterial embolization in the therapy of non-ischemic priapism—a single-center study and literature review
Source: CVIR Endovasc. 2026 Mar 18;9:30. doi: 10.1186/s42155-026-00672-0 (PMC13000060; doi:10.1186/s42155-026-00672-0)
Supplement: Supplementary file 2 — Additional file 2: Patients with follow-up in interventional radiology. [file 42155_2026_672_MOESM2_ESM.docx]

**Additional file 2** Patients with follow-up in interventional radiology

|  | **patients (n=8)** |
| --- | --- |
| Follow up time, months (median (IQR)) | 30 (15-117) |
| Age, years (median (IQR)) | 26 (19.5-38.5) |
| IIEF-5 score post-intervention (median (IQR)) | 23 (16.5-25) |
| New ED after the procedure (n (%)) | 0 (0) |
| Constant ED after the procedure (n (%))   - None - Mild - Mild to moderate - Moderate - Severe | 4 (50)  2 (25)  1 (12.5)  1 (12.5)  0 (0) |

IQR interquartile range; IIEF International Index of Erectile Function; ED erectile dysfunction. Data are presented as median (IQR) or n (%).
